# Supplementary material for: Magnetic Resonance Imaging Findings Corresponding to Vasculitis as Defined via [18F]FDG Positron Emission Tomography or Ultrasound
Source: Diagnostics (Basel). 2023 Nov 29;13(23):3559. doi: 10.3390/diagnostics13233559 (PMC10706336; doi:10.3390/diagnostics13233559)
Supplement: Supplementary file 1 [file diagnostics-13-03559-s001.zip › diagnostics-2706936-supplementary.pdf]

diagnostics

Supplementary Materials: Magnetic Resonance Imaging Findings Corresponding to Vasculitis as Defined via [^{18}F]FDG Positron Emission Tomography or Ultrasound

Table S1. Patient characteristics at diagnosis.

Characteristics	Patients (N=12)
Age, years	72.1 (65.5–74.2)
Female	7 (58%)
ESR, mm/h	64.0 (39.5–78.3)
CRP, mg/dl	40.6 (12.5–101.7)
Leukocytes, G/l	9.9 (7.1–11.5)
Thrombocytes, G/l	327.0 (263.2–462.8)
Fever	2 (17%)
Headache	5 (42%)
Jaw claudication	5 (42%)
Scalp tenderness	3 (25%)
Polymyalgic symptoms	5 (42%)
Tenderness of the temporal artery	3 (25%)
Stroke	1 (8%)
Vision loss	1 (8%)
Hypertension	3 (25%)
Diabetes mellitus	2 (17%)
Dyslipidaemia	5 (42%)
Smoking	6 (50%)
Coronary artery disease	3 (25%)
Cerebrovascular disease	2 (16%)
Peripheral artery disease	1 (8%)

Abbreviations: CRP=C-reactive protein; ESR=erythrocyte sedimentation rate; Categorical variables are shown as numbers with percentages and continuous variables as medians with interquartile ranges.

Table S2. MRI sequence protocol.

#	Name	Orientation	TR (ms)	TE (ms)	Matrix	FOV (mm^2)	Respiratory motion compensation
1	T2w HASTE	Coronal	600	30	256 x 320	40 x 480	Breath hold
2	T2w HASTE	Transverse	500	30	240 x 320	380 x 380	Breath hold
3	bSSFP	Transverse	295	1.2	208 x 256	293 x 360	Breath hold
4	T2w BLADE	Transverse	6200	119	256 x 256	350 x 350	Respiratory triggering
5	T1w VIBE Dixon pre contrast	Transverse 3D	6.7	2.4/4.8	180 x 320	300 x 400	Breath hold
6	DW-EPI b50/800	Transverse	6760	60	112 x 140	344 x 430	Free breathing
7	GRASP dynamic	Transverse 3D	3.3	1.5	256 x 256	360 x 360	Free breathing
8	T1w VIBE Dixon post contrast	Transverse 3D	6.7	2.4/4.8	180 x 320	300 x 400	Breath hold

Abbreviations: BLADE=proprietary name for periodically rotated overlapping parallel lines with enhanced reconstruction (PROPELLER) in MRI systems from Siemens Healthcare; bSSFP=balanced Steady-State Free Precession; DW-EPI=Diffusion Weighted Echo Planar Imaging; FOV=field-of-view; GRASP= Golden-angle RAdial Sparse Parallel imaging; HASTE=Half-Fourier Acquisition Single-shot Turbo spin Echo imaging; VIBE=Volumetric Interpolated Breath-hold Examination.
